# The Spectrum of Paediatric Intestinal Obstruction in Kenya

**DOI:** 10.11604/pamj.2016.24.43.6256

**Published:** 2016-05-10

**Authors:** Philip Blasto Ooko, Patricia Wambua, Mark Oloo, Agneta Odera, Hillary Mariko Topazian, Russell White

**Affiliations:** 1 Department of Surgery, Tenwek Hospital, Bomet, Kenya

**Keywords:** Intestinal obstruction, children, Kenya, ascariasis, intussusception

## Abstract

**Introduction:**

Intestinal obstruction (IO) occurs when there is impedance to the flow of intestinal contents due to a congenital or acquired pathology, and is a common paediatric surgical emergency. This study aimed to assess the pattern and outcome of paediatric IO in western Kenya.

**Methods:**

A retrospective review of all recorded cases of mechanical IO in patients aged 15 years or below admitted at Tenwek Hospital between January 2009 and December 2013.

**Results:**

The cohort included a total of 217 children (130 boys and 87 girls). The mean age was 6.7 years (range: newborn-15 years), with most (65, 30%) cases aged 1-3 years. Vomiting (161, 74.2%), abdominal pain (152, 70%), abdominal tenderness (113, 52.1%), constipation (111, 51.2%), and abdominal distension (104, 47.9%) were the predominant signs and symptoms. The most common causes of IO were ascariasis (96, 44.2%), adhesions (34, 15.7%), and intussusception (30, 13.8%). Intussusception was the leading cause of IO in children aged ≤ 1 year, ascariasis in children aged 1-5 and 6-10 years, and adhesions in children aged 11-15 years. Operative management was undertaken in 120 (55.3%) cases with 39 (32.5%) of these having gangrenous bowel. The overall mortality rate was 5%.

**Conclusion:**

The most common causes of mechanical bowel obstruction in this series were ascariasis, adhesions, and intussusception. Ascariasis remains a significant cause of paediatric IO in this region, thus public education, improved sanitation and deworming campaigns may be helpful in reducing the worm burden.

## Introduction

Intestinal obstruction (IO) occurs when there is impedance to the flow of intestinal contents due to a congenital or acquired pathology. It is a common paediatric surgical emergency and can be associated with significant mortality, especially when associated with bowel gangrene [1–3]. The pattern of IO in children in the tropics, Kenya included, is buried within the wider spectrum of IO people of all ages [2]. Age, environmental and social factors may play a role in the spectrum of IO aetiology [4, 5]. This study was conducted to ascertain the etiology, presentation, management, and outcomes of IO in children in the region and to compare these findings with those of other studies.

## Methods

This was a retrospective review of all children aged 15 years and below managed for mechanical IO at Tenwek Hospital, in Bomet Kenya between January 2009 and December 2013. Tenwek is a 320 bed hospital that acts as a referral centre for the south-western region of rural Kenya, which supports a population of over 800,000 people. Data were extracted and analysed including patient age, sex, presenting signs and symptoms, plain X-ray findings, management, complications and duration of hospitalisation. Cases involving functional IO, or with unclear diagnosis were excluded.

## Results

A total of 217 children (130 boys and 87 girls) were admitted with acute mechanical IO during the study period. The mean age was 6.7 years (range: newborn-15 years), with peak incidence in noted in children aged 1-3 years (65, 30%) (Table 1). The median symptom duration was 3 days (range 4 hours-14 days), while a history of prior laparotomy was noted in 36 (16.6%) cases. Vomiting (161, 74.2%), abdominal pain (152, 70%), abdominal tenderness (113, 52.1%), constipation (111, 51.2%), abdominal distension (104, 47.9%), and peritonitis (25, 11.5%) were the predominant signs and symptoms. An upright plain abdominal X-ray was obtained in most patients, but the results were recorded in only 158 (72.8%) cases. The main positive findings were multiple air fluid levels and dilated loops of small and large bowel. Overall, the most common causes of bowel obstruction were ascariasis (96, 44.2%), adhesions (34, 15.7%), and intussusception (30, 13.8%) (Table 2). Operative management was undertaken in 120 (55.3%) where bowel gangrene was noted in 39 cases, with the majority (18, 46.2%) being secondary to intussusception (Table 3). A total of 18 morbidities were noted in 15 (12.5%) patients in the group undergoing laparotomy, with the most common morbidity being surgical site infection in 7 cases, enterocutaneous fistula in 2 cases and fascial dehiscence in 2 cases. Six (5%) patients died, all from severe sepsis secondary to gangrenous bowel from intussusception (n = 3) or malrotation with midgut volvulus (n = 3), thus the mortality rates in patients with intussusception and, malrotation with midgut volvulus were 10% and 42.9% respectively. The mean duration of stay was 6.0 days (range 1-30). **Specific etiologies of IO:** anorectal malformation was the predominant cause of IO in neonates, most of whom presented within a few days of birth, and were successfully managed surgically. Intussusception was the leading cause of IO in children aged ≤ 1 year, and the second most common cause of IO in children between the ages of 1 and 5 years (Table 4). The median duration of symptoms for patients with intussusception was 3 days (range 1-14) with only 5 (16.7%) cases presenting within one day of symptom onset. Nonoperative reduction was undertaken in two (6.6%) cases and successful in one. Surgery was performed in 29 (96.7%) cases, with gangrenous bowel noted in 18 (62.1%) cases. Operative procedures included resection and anastomosis (18, 62.1%), manual reduction (10, 34.5%) and resection and stoma (1, 3.4%). The median duration of stay was 6 days (range 1-25). Ascariasis was the most common cause of IO in children aged 1-5 and 6-10 years. The median symptom duration for those admitted with ascariasis was 3 days (range 1-8), and the mean age was 5.5 years (range 1-15). Operative intervention to relieve the obstruction was performed in 30 (31.2%) cases, consisting of enterotomy and worm extraction in 22 (73.3%) cases with viable bowel, and resection and anastomosis in the 8 (26.7%) cases with gangrenous bowel. The mean duration of stay was 4.6 days (range 1-16). Adhesive bowel disease was the predominant cause of IO in children between the ages of 11 and 15 years and the second most common cause of IO in those between the ages of 6 and 10 years. The adhesions were post-operative in 27 (79.4%) cases and post-inflammatory in 7 (20.6%) cases. The median duration of symptoms was 3 days (range 1-9), and the majority (38.2%) of the cases were aged between 11 and 15 years. Management was operative in 17 (50%) cases, mainly involving lysis of adhesions. Bowel resection was performed in the two cases with gangrenous bowel. The median duration of stay was 6 days (range 1-25).

**Table 1:** Distribution of cases by age (n = 217)

Age (years)	Cases	Percentage (%)
< 1	32	14.7
1-3	65	30
4-6	37	17
7-9	35	16.1
10-12	19	8.8
13-15	29	13.4

**Table 2:** Distribution of cases by etiology and management (n = 217)

Etiology	Cases	Management	
		Operative	Non-operative
Ascariasis	96	30	66
Adhesions	34	17	17
Intussusception	30	29	1
Small bowel volvulus	16	16	-
Anorectal Malformations	9	9	-
Malrotation	7	7	-
Fecal impaction	4	0	4
Hirschsprung's disease	4	4	-
Other	17	8	9
Total	217	120	97

**Table 3:** Etiology of IO cases with bowel gangrene (n = 39)

Etiology	Number of cases	Percentage (%)
Intussusception	18	46.2
Ascariasis	8	20.5
Small bowel volvulus	7	17.9
Malrotation	2	5.1
Adhesions	2	5.1
Other	2	5.1

**Table 4:** Common etiologies of IO cases by age group (n = 217)

Age group	Etiology	Number of cases (percentage)
≤1 month (n = 13)	Anorectal malformations	7 (53.8%)
	Malrotation	4 (30.8%)
>1 month-1 year (n = 19)	Intussusception	16 (84.2%)
	Hirschsprung's disease	2 (10.5%)
1-5 years (n = 92)	Ascariasis	59 (64.1%)
	Intussusception	11 (12%)
6-10 years (n = 56)	Ascariasis	29 (51.8%)
	Adhesions	11 (19.6%)
11-15 years (n = 37)	Adhesions	13 (35.1%)
	SBV	10 (27%)

## Discussion

Intestinal obstruction is a common pediatric surgical emergency whose etiologies vary mainly with patient age [5]. Anorectal malformation is the most common cause of IO in neonates at 35-39%, followed by hirschprung's disease at 11-23% [6, 7]. Similar findings were noted in this series, with the slightly higher proportions due to the lower number of cases. Intussusception has been reported to be not only the most common cause of IO in infants [8, 9], but also the most common cause of IO in children overall, responsible for 22-46% of all cases of IO [1, 2, 4, 5]. In this study, intussusception was the most common cause of IO in infants. Similar to other studies from developing countries, most of the cases of intussusception in this series were managed surgically [1, 2, 5, 8–12]. The predominant operative management is due to delayed patient presentation with concern for gangrenous bowel, predominant clinical diagnosis, with a significant proportion of cases being confirmed at laparotomy and lack of around-the-clock radiologic facilities and personnel to perform nonsurgical reduction [2, 11, 12]. This argument is underscored in this series where the median durationof presentation was 3 days, with gangrenous bowel noted in 62.1% of the cases. The mortality rate of cases with intussusception in this series at 10%, is similar to the reported mortality rate of 5-28% in series form developing countries [8, 10–12].

Ascariasis was the leading overall cause of IO in this series, accounting for 44.2% of all cases of IO; 64.1% of IO cases in children aged 1-5 years, and 51.8% of all cases in children aged 6-10 years. Similarly, two studies from Kashmir reported ascariasis to be the cause of IO in 63-77% of the study cohort [13, 14]. Ascariasis is a common infection in the tropics, and is associated with poor hygiene and low socioeconomic conditions that are more prevalent in rural areas such as western Kenya [14]. The incidence of IO estimated to be 1 in 500 infected children [15], with peak age between 2 and 10 years [16–18]. While most cases can be managed non-operatively, the proportion of patients undergoing operative intervention has been reported to be between 8% and 26% [15–18]. Main procedures performed include the milking of worms into the colon (48-71%), enterotomy for worm extraction (13-49%), and resection and anastomosis in the face of bowel gangrene or perforation (14-26%)[16–18]. The reported overall mortality is 0-1%, and 0-6% in those undergoing operative management [15–18]. Adhesions have been reported to be the second most common cause of IO, accounting for 10-16% of all cases of IO in series from developing countries [1, 4, 13]. Most are post-operative, while operative intervention is required in 53-66% of cases [4,14, 19]. Similar findings were noted in this series. The clinical presentation of the patients in this study is similar to studies performed in developing countries, with abdominal pain at 60-89%, vomiting at 63-85%, abdominal distension at 43-82 and constipation at 35-70%, being the most commonly reported signs and symptoms [1, 5, 14]. The overall mortality rate in this series at 3.2%, is comparable to the reported mortality rate of 2%-11% from similar series conducted in developing countries [1, 2, 4, 5].

## Conclusion

The most common causes of mechanical IO in this series varied by age and included ascariasis, adhesions, and intussusception. Most cases of intussusception were managed operatively due to delayed presentation, predominant clinical diagnosis and presence of gangrenous bowel. The highest rates of morbidities were noted in patients with bowel gangrene due to intussusception and malrotation, thus a high index of suspicion, and early management may help to improve outcomes. Ascariasis remains a significant cause of pediatric IO in this region, thus public education and mass deworming campaigns may be helpful in reducing the worm burden, and thus the incidence of IO.

### What is known about this topic

- Intestinal obstruction (IO) is a common paediatric surgical emergency and can be associated with significant mortality, especially when associated with bowel gangrene.
- The spectrum of IO aetiology depends on the age, environmental and social factors.
- Intussusception has been reported to be the most common cause of IO in children overall.

### What this study adds

- Ascariasis was the leading overall cause of IO in this series, and the most common cause of IO in children aged 1-5 years and 6-10 years.
- Intussusception was the most common cause of IO in infants, with most cases managed surgically.
- The highest rates of morbidities were noted in patients with bowel gangrene due to intussusception and malrotation.
